# Associated factors, inequalities, and spatial distribution of the use of modern contraceptive methods among women of reproductive age in Peru: a population-based cross-sectional study

**DOI:** 10.1186/s12889-022-14629-0

**Published:** 2022-12-05

**Authors:** Ana Lucía Díaz-Alvites, Gonzalo Yrala-Castillo, Ali Al-kassab-Córdova, César V. Munayco

**Affiliations:** 1 Faculty of Health Sciences, Universidad Peruana de Ciencias Aplicadas, Lima, Peru; 2 Sociedad Científica de Estudiantes de Medicina de La Universidad Peruana de Ciencias Aplicadas (SOCIEMUPC), Lima, Peru; 3 Center of Excellence in Economic and Social Research in Health, Universidad San Ignacio de Loyola, Lima, Peru

**Keywords:** Family Planning, Contraceptive Methods, Healthcare Inequalities, Spatial Analysis, Demographic and Health Surveys, Peru

## Abstract

**Background:**

The use of contraceptive methods in Peru has remarkably increased in recent decades. Nevertheless, despite the completeness and accessibility of family planning methods, modern contraceptive methods utilization in Peru remains below the South American average. Thus, this study aimed to elucidate the factors associated with modern contraceptive use, as well as the presence of inequalities and the spatial distribution in Peruvian women aged 15–49 years in 2019.

**Methods:**

A secondary data analysis was conducted using information from the 2019 Peruvian Demographic and Health Survey. We performed descriptive statistics, bivariate analysis, and Poisson multiple regression. Inequalities were estimated through concentration curves and Erreygers’ normalized concentration index. Spatial analysis included choropleth map, Global Moran’s I, Kriging interpolation and Getis-Ord-Gi* statistic.

**Results:**

The prevalence of modern contraceptive use was 39.3% among Peruvian women of reproductive age. Modern contraceptive use was directly associated with youth (aPR 1.39), women having their first sexual intercourse before the age of 18 (aPR 1.41), and being married but not together (aPR 1.87). In addition, speaking Quechua or Aymara (aPR 0.87) and having no children (aPR 0.59) were inversely associated with utilization of modern contraceptives. We found the presence of inequalities in the use of contraceptive methods (pro-rich distribution), although the magnitude was low. Spatial analysis unveiled the presence of a clustered distribution pattern (Moran’s Index = 0,009); however, there was inter-departmental and intra-departmental heterogeneity in the predicted prevalence of the use of modern contraceptives. In addition, significant hot and cold spots were found in Peru.

**Conclusion:**

Two out of five Peruvian women of reproductive age used modern contraceptives. It was associated with younger women’s age, younger age at first sexual intercourse, being married or cohabitant, among others. No substantial inequality was found in modern contraceptive use. The prevalence was heterogeneous at the intra- and inter-departmental level. Those departments located in the south, south-east, and north-east had the lowest prevalence. Therefore, nonfinancial barriers must be tackled through multi- and cross-sectoral efforts and continue to universally provide modern contraceptives.

## Background

Family planning (FP) is the health strategy that empowers individuals to decide whether to have children, how many they want, and the spacing between pregnancies [1]. FP is facilitated through contraceptive methods (CM). It encompasses interventions that occur prior to the prescription and provision of CM, such as information dissemination, education, and counseling [2].

A CM is any method, medication, or device used to prevent pregnancy [3]. There are two types of major CM, viz., the traditional contraceptive methods (TCM) and modern contraceptive methods (MCM). There is no consensus on the definition of MCM, thus the measurement of MCM differs between studies [4]. MCM were designed to permit complete sexual freedom, and the decision to use CM is at the discretion of the individual or couple.

Various studies have highlighted the association between certain sociodemographic factors and the use of CM, such as the woman’s age, educational level, employment status, and socioeconomic status [5–10]. Soriano-Moreno DR, et al. conducted a study in Peruvian women and they found that having one or more children and having health insured children were associated with the use of highly effective contraceptive methods (HECM) [11].

Globally, almost one in two women of reproductive age used a form of CM in 2019, a slight increase compared to previous decades. However, there remains a significant unmet demand and regional gaps persist [12]. In Peru, the use of MCM has remarkably increased in recent decades [13, 14]. FP programs seek to reach the entire population under approaches of interculturality, comprehensiveness, gender equity and social inclusion. Nevertheless, despite the completeness and accessibility of FP methods due to the Ministry of Health (MINSA, from Spanish acronym) policies [2], MCM utilization in Peru remains below the South American average (68%) and the use of TCM remains high [15, 16]. In addition, the majority of Peruvian women reported having more children than desired [17].

Access to CM is a human right [18, 19]. The United Nations aims to eliminate all the unmet demands for FP by 2030. Improvements in reproductive health, including voluntary FP, can bolster economies, contribute to sustainable development, and reduce pregnancy-related costs [20]. To do so, it is essential to implement targeted strategies to reduce geographic and socioeconomic gaps in access. In Peru, there has been improvements in access and coverage of health services. However, significant inequalities remain that require resolution, especially in the most vulnerable populations [21]. By elucidating the determinants of MCM use and its geographic pattern, policymakers would redirect their policies. Otherwise, the fertility rate may increase, which entails risks to the health of children and their mothers, undermines investment in human capital, dampens economic growth, and aggravates environmental threats [22]. Therefore, we conducted this study to elucidate factors associated with the use of MCM, in addition to the magnitude of inequality and the spatial distribution among Peruvian women of reproductive age.

## Methods

### Study design and data sources

We conducted a secondary data analysis using information from the 2019 Peruvian Demographic and Health Survey (DHS). The DHS is annually conducted by the National Institute of Statistics and Informatics (INEI, from the Spanish acronym) of Peru. It has national, departmental, and area of residence representativeness. Administratively, the Peruvian territory is divided into 24 departments and one constitutional province, which are subsequently subdivided into provinces and districts. The survey design was probabilistic, two-stage, balanced, stratified, independent, and self-weighted. The 2019 survey included 36,745 households, from which 35,522 individuals were interviewed [23]. Although the Peruvian DHS collects information at the household level, it is mapped at the sampling cluster level.

### Selection criteria

The DHS includes women aged 12–49 years. However, our study included only women of reproductive age. According to the World Health Organization (WHO), women of reproductive age are those aged between 15–49 years [24]. Participants with incomplete data for the variables of interest were excluded.

### Outcome definition

The outcome variable (use of MCM) was defined according to the WHO definition [25]. It was categorized into MCM utilization and MCM non-utilization. MCM utilization included oral contraceptive pills, intrauterine device, injectables, female and male condoms, female and male sterilization, implants, lactational amenorrhea method, vaginal barrier methods, and emergency contraception pills. MCM non-utilization included traditional and folkloric methods, such as abstinence, periodic abstinence, and withdrawal, and no method use.

### Independent variables

We included 11 independent categorical variables related to social determinants of inequality, which were selected based on an extensive literature review. Age was divided into three groups: 15–19, 20–34, and 35–49 years. Natural region was categorized into coast, highlands, rainforest, and Metropolitan Lima. In addition, we included other sociodemographic variables such as residence area, marital status, education, employment status, language, wealth index, age at first sexual intercourse, number of children alive, and family members.

### Socioeconomic status

The wealth index was used as a proxy variable to socioeconomic status. The DHS does not directly measure living standard (for instance income); it is a measurement of relative socioeconomic position of a household and is based on household data of ownership and housing characteristics. Subsequently, it is calculated through principal component analysis [26, 27].

### Statistical analysis

The DHS databases were downloaded from the “Microdatos” webpage of the INEI [28]. Descriptive, bivariate, multiple regression, and inequality analyses were conducted using STATA version 16.0 (Stata Corporation, College Station, TX, US). Estimates were made by considering the complex design of the survey (strata, weights, and primary sampling units) through the *svy* module. *P*-values of < 0.05 were considered to be significant, and confidence intervals were computed to 95% (95% CI).

Descriptive analysis was performed to obtain absolute and relative frequencies. The prevalence of MCM use was estimated at national and departmental levels. Bivariate analysis was performed to evaluate the prevalence of MCM use among independent variables for which Pearson’s chi-square test was used. Prevalence ratios were estimated to evaluate the magnitude of association between independent variables and MCM use, crude (cPR), and adjusted (aPR). Consequently, the generalized linear model (glm) Poisson family log link function was used. We computed this model as it assumes adequate probability distribution, there is no numerical instability, variances are smaller, and PR is more interpretable than other measures of association [29, 30].

### Concentration curve and index

The *lorenz* and *conindex* commands were used for the analysis of inequalities [31, 32]. The magnitude of wealth inequality in use of MCM was estimated through concentration curves (CC) and concentration index (CI). CC represents the distribution of health among the cumulative proportions of a specific population classified according to their socioeconomic level: from the poorest to the richest. This curve has the distribution of women surveyed ordered from the lower to higher socioeconomic level on its X-axis and the health variable (in our case, the use of MCM) on its Y-axis. If the proportion of health was equally distributed among the population based on their income, a 45° diagonal would be generated, and the CI would equal zero. A deviation of the curve to either side indicates the existence of inequality. The separation of the curve from the diagonal generates an area under the curve (AUC), which will be the value of the CI. A positive value of the CI (curve below the diagonal) implies that inequality in access to health is more concentrated among the rich groups, and a negative CI value (curve above the diagonal) implies greater inequality among poor groups [33]. CI values close to zero represent the existence of very little inequality, whereas CI values close to + / − 1 indicate the existence of greater inequality [34]. The greater the AUC (represented by CI), the greater the inequality.

Considering that MCM use is a binary variable, Erreygers’ normalized concentration index (ECI) was used in our study instead of CI. This is mathematically depicted below.

$$ECI\left(h\right)= \frac{1}{n}\sum_{i=1}^{n}4{h}_{i}({2R}_{i}-1)$$

where: *n* represents the sample size, *h*_*i*_ is the binary outcome of interest for person *i* (with limit values of 0 and 1), and *Ri* is the individuals rank by wealth index. Weighted ECI standardizes the uncorrected index by adjusting the CI to allow for the bounded nature of the variable under study. Therefore, certain axiomatic properties for an inequality index (transfer, level independence, cardinal invariance, and mirror) are satisfied [32, 35–37].

### Spatial analysis

All spatial analyses were performed in ArcGIS version 10.8 (ESRI, Redlands, CA, US). A choropleth was plotted to represent the regional prevalence of MCM utilization. To evaluate the spatial autocorrelation of the outcome variable, Global Moran’s I was calculated. It ranges from -1 to 1. A positive value implies a clustered pattern, a negative value implies a dispersed pattern, and cero implies a random pattern. In addition, we conducted ordinary Kriging interpolation analysis to predict the prevalence of MCM utilization in unsampled locations. Furthermore, we evaluated the presence of hot and cold spots through Getis-Ord-Gi* statistic.

## Results

### Characteristics of the study population

A total of 33,311 women aged 15–49 years were included in the analysis. Their mean age was 31.29 years (SD: 9.99). Almost half of the participants were aged between 20–34 years (43.9%). The majority of participants were from Metropolitan Lima (43.2%) and lived in urban areas (82.6%), and over half were currently married or living with their partner (55.3%). The majority of participants had reached the secondary level education (44.9%), was employed (71.5%), spoke Spanish (94.5%) and had a middle wealth index (21.7%). The majority of women had experienced their first sexual intercourse before the age of 18 years (40.5%), had 1–2 living children (42.5%), and had ≤ 4 members in their family (54.4%) (Table 1).

**Table 1:** Descriptive and bivariate analysis of MCM use among Peruvian women of reproductive age

Population characteristics	Variable categories	Frequency n (%)	Modern contraceptive methods utilization		*p*-value*
			Yes (%)	No (%)	
Age	15–19	4,668 (15.7)	11.8	88.2	< 0.001
	20–34	16,994 (43.9)	45.3	54.7	
	35–49	11,627 (40.4)	43.5	56.5	
Natural region	Coast	11,216 (27.9)	37.7	62.3	< 0.001
	Highlands	10,005 (20.3)	35.6	64.4	
	Rainforest	6,370 (8.6)	44.7	55.3	
	Metropolitan Lima	5,698 (43.2)	41.0	59.0	
Residence area	Urban	23,859 (82.6)	39.6	60.4	0.1049
	Rural	9,430 (17.4)	38.0	62.0	
Marital status	Never married	7,418 (31.4)	15.0	85.0	< 0.001
	Married/Cohabitant	21,907 (55.3)	55.6	44.4	
	Married but not together	3,964 (13.4)	29.4	70.6	
Education	No education/Primary	6,651 (15.9)	40.5	59.5	0.0043
	Secondary	15,671 (44.9)	37.5	62.5	
	Higher	10,967 (39.2)	41.0	59.0	
Employment status	Unemployed	10,416 (28.5)	36.7	63.3	0.0013
	Employed	22,873 (71.5)	40.4	59.6	
Language	Quechua/Aymara	2,649 (4.7)	31.6	68.4	0.0003
	Spanish	30,161 (94.5)	39.7	60.3	
	Other native and/or foreign languages	479 (0.8)	36.7	63.3	
Wealth index	Poorest	8,671 (16.5)	35.5	64.5	0.0104
	Poor	8,618 (20.2)	41.1	58.9	
	Middle	6,815 (21.7)	38.9	61.1	
	Rich	5,290 (21.1)	39.6	60.4	
	Richest	3,895 (20.5)	40.9	59.1	
Age at first sexual intercourse	Never had sexual intercourse	3,620 (15.5)	0.1	99.9	< 0.001
	< 18	15,919 (40.5)	50.4	49.6	
	18–24	12,249 (38.4)	44.6	55.4	
	≥ 25	1,501 (5.6)	31.9	68.1	
Number of children alive	Has no children	6,991 (33.9)	16.7	83.3	< 0.001
	1–2	16,382 (42.5)	49.3	50.7	
	3–4	7,586 (18.6)	56.4	43.6	
	≥ 5	2,330 (5.0)	44.9	55.1	
Family members	≤ 4	15,581 (54.4)	38.3	61.7	0.0246
	> 4	17,708 (45.7)	40.6	59.4	

*Pearson’s chi-squared test

**Fig. 1:**
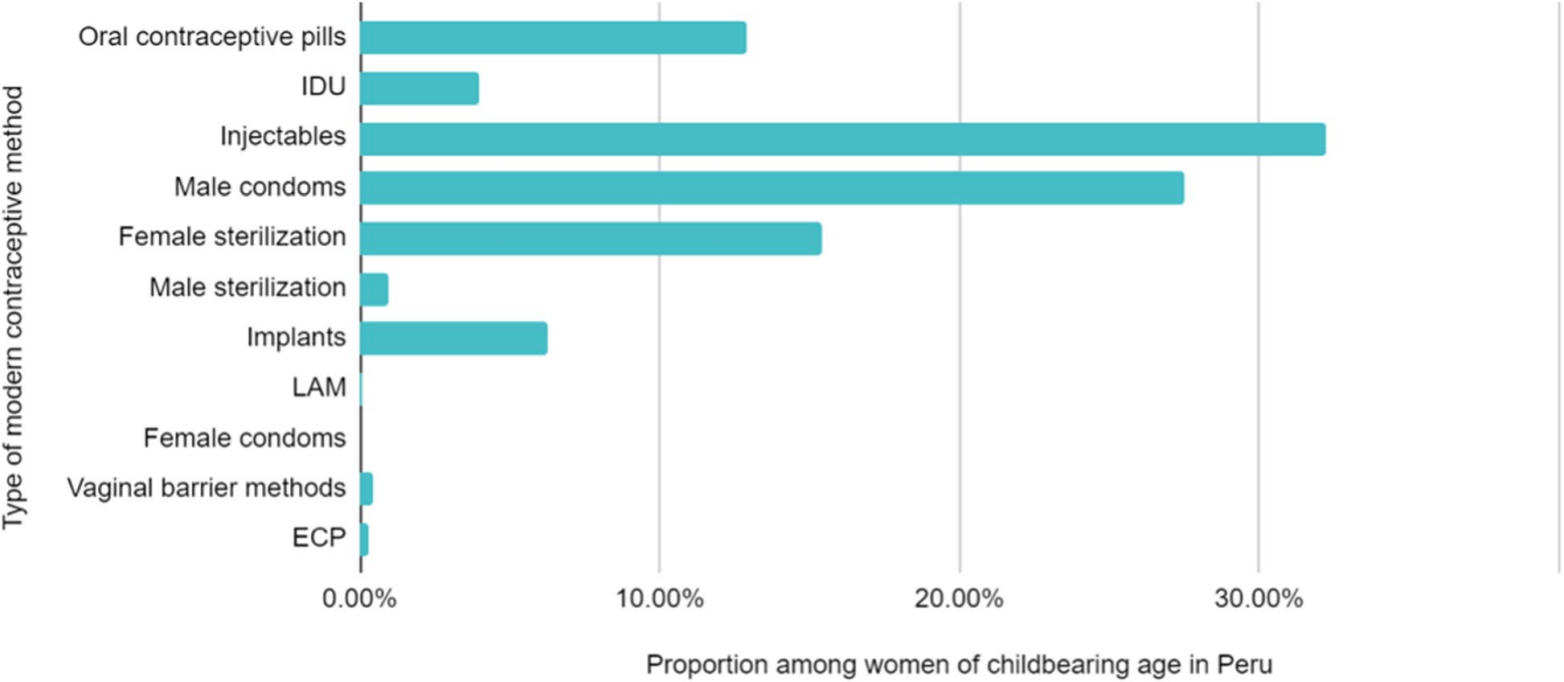
Proportion of MCM use among Peruvian women of reproductive age. IDU: Intrauterine device. LAM: Lactation amenorrhea method. ECP: Emergency contraceptive pill

In 2019, 39.3% of Peruvian women of reproductive age used MCM. The most used MCM were injectables (32.3%), male condoms (27.5%), female sterilization (15.4%), and oral contraceptive pills (12.9%) (Fig. 1)

### Bivariate analysis

Table 1 shows the prevalence of MCM use according to each independent variable. MCM were used by 11.8% of women aged 15–19 years. In all the natural regions of Peru, the prevalence of MCM use was low (37.7% on the coast, 35.6% in the highlands, 44.7% in the rainforest, and 41% in Metropolitan Lima). Regarding marital status, the majority of women who used MCM were married or lived with their partner (55.6%). Regarding education, women with a higher degree of education primarily used MCM (41%). In addition, MCM utilization was higher among employed women (40.4%). Furthermore, those speaking Spanish used more MCM (39.7%), than those speaking Quechua/Aymara (31.6%). In addition, women who had a poor wealth index primarily used these methods (41.1%), and MCM was higher among those who had their first sexual intercourse before the age of 18 years (50.4%) and among women who had 3–4 living children (56.4%). Finally, women who had over four members in their family also had higher MCM use (40.6%). Aside from residence area, all of these variables showed statistically significant differences.

### Multiple regression analysis

We performed a multiple regression analysis to identify independent predictors of MCM use among Peruvian women of reproductive age. Being young (aged 15–19 years) was associated to a 39% greater chance (aPR 1.39; 95% CI: 1.20–1.59) of MCM utilization than older women (aged 35–49 years). Similarly, married women, or those who lived with their partner had 87% more likelihood of using MCM than women who were married but not together (aPR 1.87; 95% CI: 1.69–2.06). Having higher education was associated with 23% higher probability (aPR 1.23; 95% CI: 1.13–1.34) of using MCM, compared to having no education or primary. Other sociodemographic variables associated with higher MCM use were richest wealth index (aPR 1.33; 95% CI: 1.19–1.48) and having had their first sexual intercourse at < 18 years of age (aPR 1.41; 95% CI: 1.22–1.62). However, certain variables demonstrated a protective effect concerning the use of MCM. Women living in the highlands were 13% less likely (aPR 0.87; 95% CI: 0.82–0.93) to use MCM than those living in Metropolitan Lima. Being employed was associated with 0.93 times less likelihood (aPR 0.93; 95% CI: 0.89–0.98) of using MCM than being unemployed. Similarly, Quechua or Aymara speakers were 13% less likely (aPR 0.87; 95% CI: 0.79–0.95) to use MCM compared to those who speak Spanish. In addition, having 1–2 children alive was associated with 0.88 times less likelihood (aPR: 0.88; 95% CI: 0.79–0.98) of using MCM compared with having over 4 children (Table 2).

**Table 2:** Multiple regression analysis of MCM use among Peruvian women of reproductive age

Population characteristics	Crude analysis		Adjusted analysis	
	(c)PR^a^	95% CI	(a)PR^b^	95% CI
Age				
15–19	0.27	0.24–0.32***	1.39	1.20–1.59***
20–34	1.04	0.99–1.10	1.30	1.23–1.37***
35–49	Ref	Ref	Ref	Ref
Natural region				
Coast	0.92	0.86–0.98*	0.91	0.86–0.96**
Highlands	0.87	0.81–0.93***	0.87	0.82–0.93***
Rainforest	1.09	1.02–1.17*	0.96	0.90–1.03
Metropolitan Lima	Ref	Ref	Ref	Ref
Residence area				
Urban	1.04	0.99–1.10	0.96	0.91–1.02
Rural	Ref	Ref	Ref	Ref
Marital status				
Never married	0.51	0.44–0.58***	1.15	0.99–1.33
Married/Cohabitant	1.89	1.71–2.09***	1.87	1.69–2.06***
Married but not together	Ref	Ref	Ref	Ref
Education				
No education/Primary	Ref	Ref	Ref	Ref
Secondary	0.93	0.87–0.99*	1.12	1.05–1.19**
Higher	1.01	0.94–1.09	1.23	1.13–1.34***
Employment status				
Unemployed	Ref	Ref	Ref	Ref
Employed	1.10	1.04–1.17***	0.93	0.89–0.98**
Language				
Quechua/Aymara	0.80	0.72–0.88***	0.87	0.79–0.95**
Spanish	Ref	Ref	Ref	Ref
Other native and/or foreign languages	0.92	0.70–1.22	0.83	0.62–1.11
Wealth index				
Poorest	Ref	Ref	Ref	Ref
Poor	1.16	1.09–1.23***	1.15	1.08–1.23***
Middle	1.10	1.02–1.18**	1.17	1.08–1.28***
Rich	1.12	1.03–1.21**	1.22	1.11–1.34***
Richest	1.15	1.06–1.26***	1.33	1.19–1.48***
Age at first sexual intercourse				
Never had sexual intercourse	0.00	0.00–0.01***	0.00	0.00–0.01***
< 18	1.58	1.36–1.83***	1.41	1.22–1.62***
18–24	1.40	1.20–1.62***	1.29	1.12–1.49***
≥ 25	Ref	Ref	Ref	Ref
Number of children alive				
Has no children	0.37	0.32–0.43***	0.59	0.50–0.70***
1–2	1.10	1.00–1.20*	0.88	0.79–0.98*
3–4	1.26	1.15–1.38***	1.09	0.99–1.20
≥ 5	Ref	Ref	Ref	Ref
Family members				
≤ 4	Ref	Ref	Ref	Ref
> 4	1.06	1.01–1.12*	1.01	0.96–1.07

(c) PR: crude prevalence ratio. (a) PR: adjusted prevalence ratio

*p-value <0.05, **p-value<0.01, ***p-value<0.001

### Inequalities analysis

**Fig. 2:**
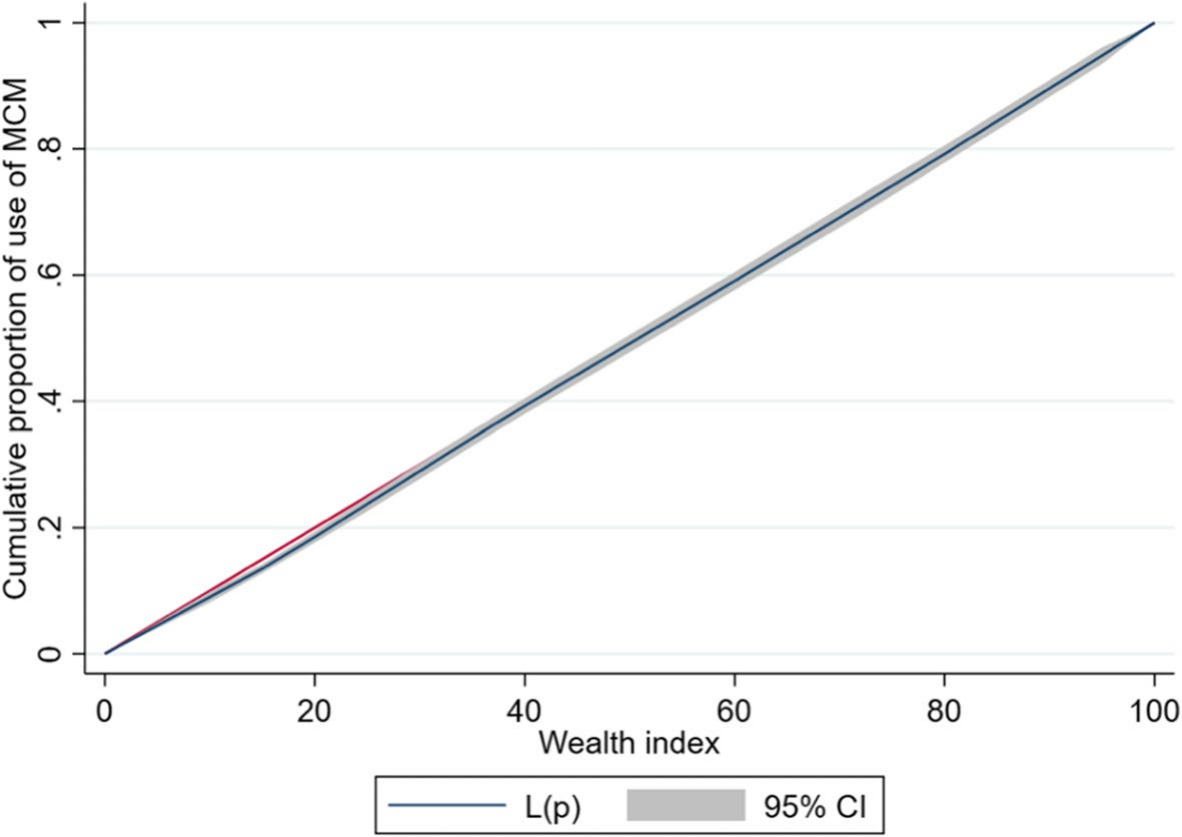
Concentration curve of wealth-related inequalities for MCM use among Peruvian women of reproductive age

**Fig. 3:**
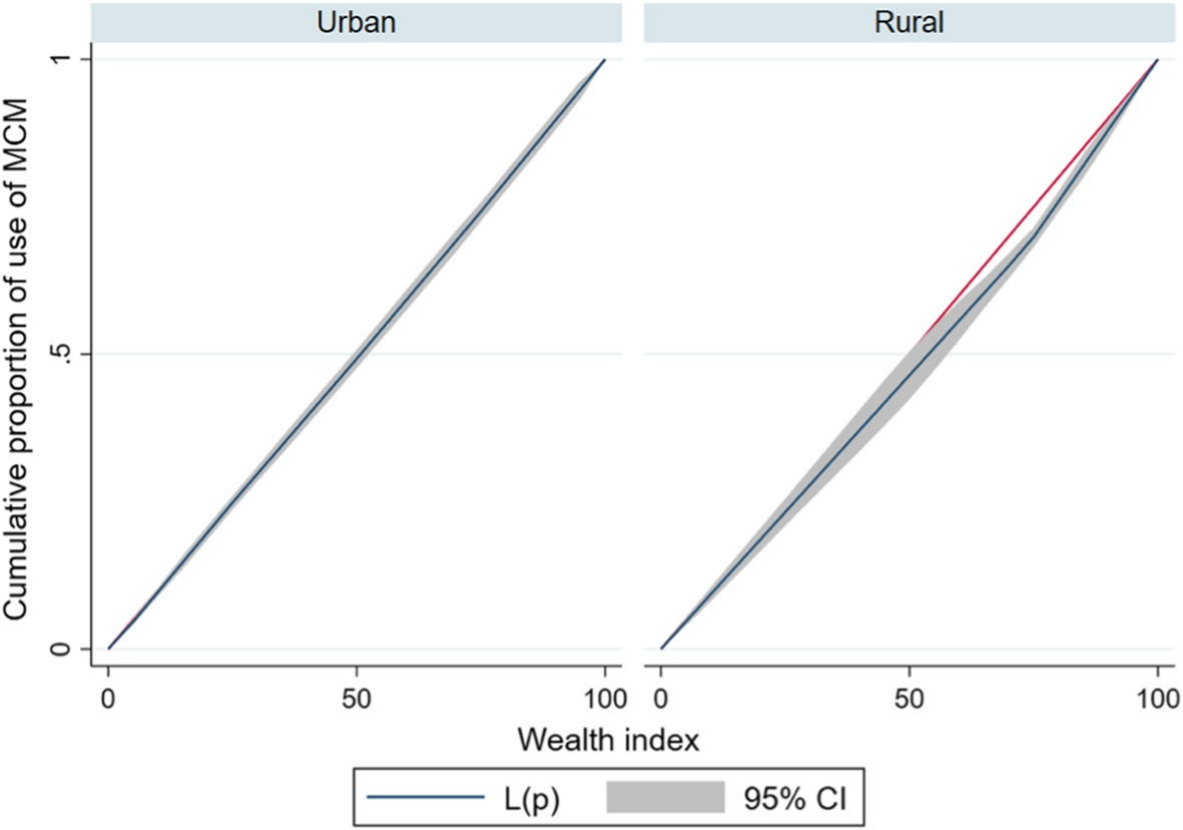
Concentration curves of wealth-related inequalities for MCM use among Peruvian women of reproductive age stratified by residence area

The prevalence of MCM use indicated a pro-rich distribution among Peruvian women, albeit low in magnitude (ECI = 0.026). Moreover, the inequalities in MCM use were higher in rural areas (ECI = 0.079) than in urban areas (ECI = 0.015). Similarly, the concentration curve indicated that the distribution of MCM use was concentrated in rich households (Figs. 2 and 3).

### Spatial analysis

**Fig. 4:**
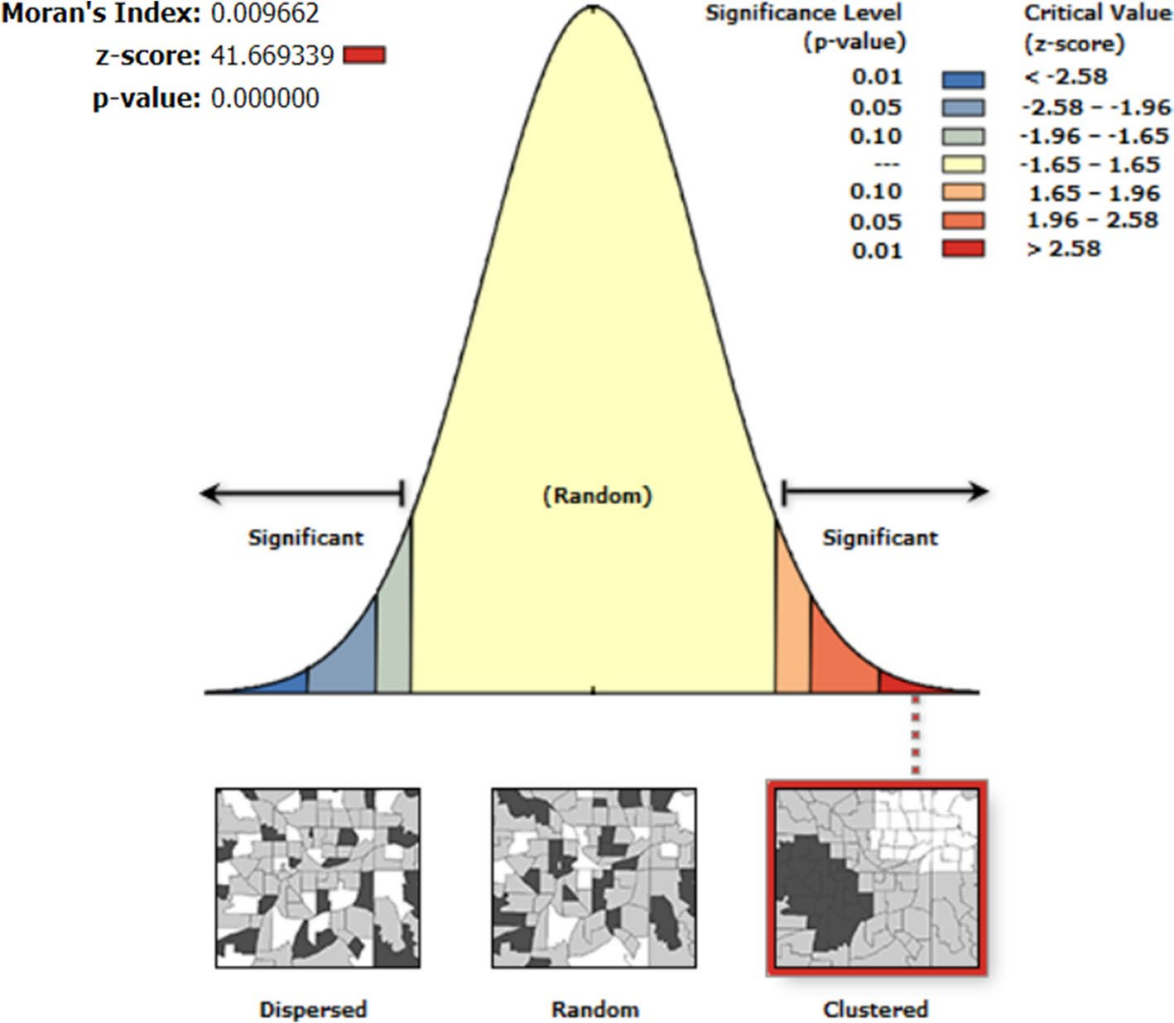
Spatial autocorrelation (Global Moran’s I) of MCM use among Peruvian women of reproductive age

The spatial distribution of MCM use in Peruvian women had a clustered pattern (Moran’s Index = 0,009, *p*-value < 0.001; Fig. 4).

Given the z-score of 41.6693389136, there is a less than 1% likelihood that this clustered pattern could be the results of random chance.

**Fig. 5:**
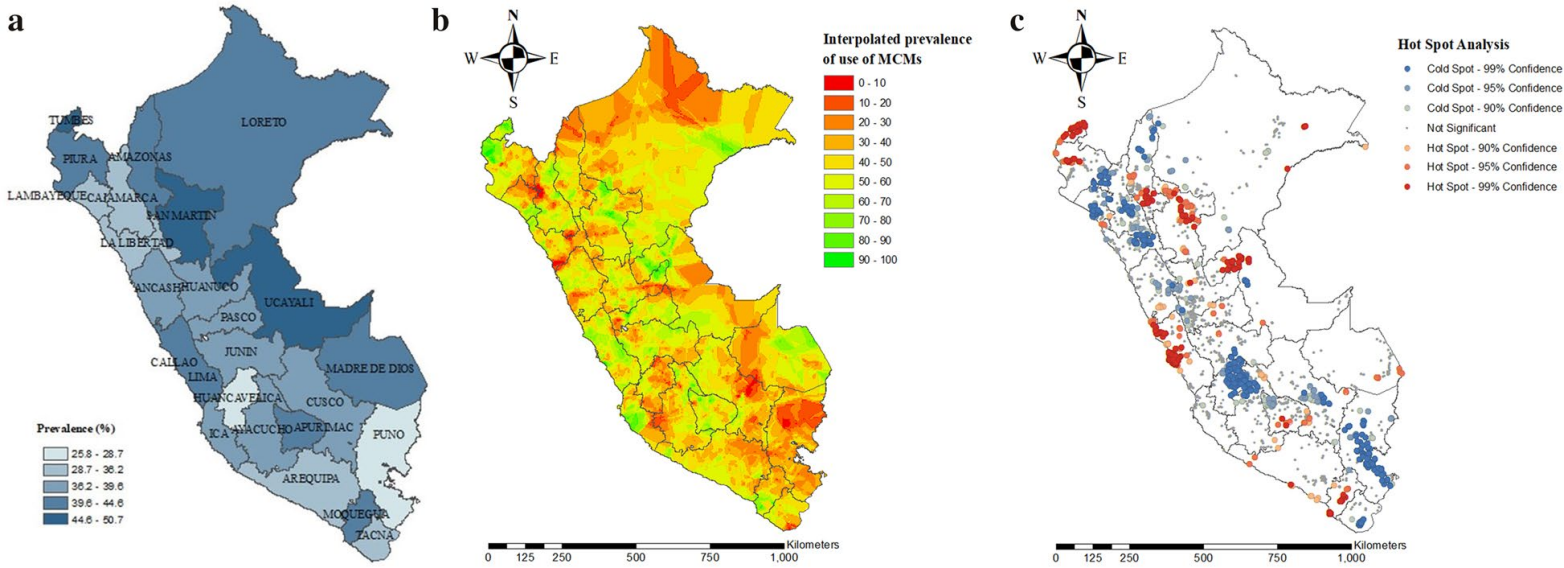
Spatial analysis of MCM use among Peruvian women of reproductive age. **5a. **Departmental prevalence of MCM use. **5b**. Kriging interpolation of MCM use. **5c**. Hot spot analysis (Getis-Ord-Gi*) of MCM use

The choropleth map represents the prevalence of MCM utilization at the departmental level. The departments with the highest prevalence of MCM use were Tumbes (50.7%), San Martín (48.4%), and Ucayali (46%). Puno (25.8%), Huancavelica (28.7%), and Tacna (33.1%) reported the lowest use of MCM (Fig. 5a).

Kriging interpolation analysis depicts the predicted prevalence of MCM use. The prevalence increases from red (low prevalence) to green-colored (high prevalence) areas. Those departments located in the south, south-east, and north-east had the lowest predicted prevalence of use of MCM (Fig. 5b).

Hot spot (Getis-Ord-Gi*) analysis shows red and blue points, which represent a more intense clustering of high and low proportion of MCM use, respectively. A high proportion of MCM use was found in Tumbes, Lima, Ucayali, Amazonas, Moquegua, and San Martin regions. Meanwhile, a low proportion of use of MCM was found in Puno, Huancavelica, Cajamarca, Lambayeque, Tacna, and La Libertad (Fig. 5c).

## Discussion

### Main findings

Several sociodemographic factors were associated with MCM utilization, although the prevalence was low and with high variability between departments among Peruvian women of reproductive age. The strongest association was found with women’s age, age at first sexual intercourse, marital status, and language, even after adjusting for multiple potential cofounders. Other associated variables were natural region, education, wealth index, employment status, and number of living children. Moreover, the most used MCM were injections, male condoms, and female sterilization. Regarding the CI, our study revealed the presence of inequalities in the use of MCM (pro-rich distribution), although the magnitude was low. However, spatial analysis unveiled the presence of a clustered distribution pattern (albeit low in magnitude), but there was inter-departmental and intra-departmental heterogeneity in the predicted prevalence of the MCM use. In addition, we found significant hot and cold spots of MCM utilization across Peru.

### Comparison with previous studies

Approximately two out of five Peruvian women of reproductive age use MCM. Although the prevalence has increased, it is below the South American average (68.2%) and that of other Latin American countries, such as El Salvador (66.8%), the Dominican Republic (67.1%), Nicaragua (68.8%), and Costa Rica (73.9%) [6, 15, 38]. However, this may be due to the large indigenous population in Peru. A study reported that the use of TCM was higher in countries with larger indigenous populations while the use of MCM was lower [38], and Peru has the highest proportion of TCM use in Latin America [6]. Other explanatory factors include limited access to FP due to geographic and language differences, or a different stage of the demographic transition.

Soriano-Moreno DR, et al. investigated the factors associated with the use of HECM among Peruvian women of reproductive age [11]. They reported a prevalence of 29.9% concerning the use of HECM, whereas we reported a prevalence of MCM utilization of 39.3%, as our outcome definition included more FP methods. Similar associations were found in both studies. However, our study included a spatial and inequality analysis, and they did not include language, age at first sexual intercourse, and the number of family members as exposure variables. In addition, our outcome variable is broader as it encompasses more CM (beyond those that are highly effective). Finally, the database used in our study is more updated: at the end of 2017, the FP Technical Standard was implemented in Peru, which could have significantly impacted the use of MCM [2].

### Factors associated with MCM use

Centralization has been problematic in Peru for decades. Women living in the coast and highlands were less likely to use MCM than women living in Metropolitan Lima (the capital of Peru). The Peruvian health system is fragmented and segmented, with large gaps [39, 40]. For instance, Lima has the largest number of health facilities and physicians per inhabitant countrywide [41]. Centralization fuels health inequalities, therefore, the deconcentration of health resources is necessary.

Younger women were more likely to use MCM, which may be due to the recent implementation of FP policies that include health guidance and counseling towards adolescents nationwide [42, 43]. However, a number of previous studies have established that older women are typically associated with greater use of MCM . Therefore, data regarding the prevalence of MCM utilization between age groups are controversial [10, 44–47].

Education is essential to increase the use of MCM. Those women with a higher degree were more prone to use MCM, which aligns with several previous studies [5, 6, 10, 48]. Indeed, those with lower educational level are the most affected by existing inequalities in Peru [21].

Having their first sexual intercourse at below 18 years of age was associated with greater use of MCM. Similar results were found in Ethiopia [49]. This could be because younger women are more sexually active than older women and are economically dependent (in most cases). Economic independence is important because having children implies a significant financial burden. Interestingly, sex education (if provided before first experience of sexual intercourse), which is included in the Peruvian school’s curriculum, protects youth from having sex at an early age [50].

Both marital status and number of children were associated with the use of MCM. Women who were married or lived with their partner had more chances to use MCM. This aligns with the results from a study from Uganda, which found that married adolescents were more likely to use MCM than unmarried adolescents. It would have been interesting to analyze the level of education of the partner and its influence on MCM use in our study, as undertaken by the study in Uganda [51]. Nevertheless, this variable was excluded from our study as there were missing data in the Peruvian DHS.

Quechua and Aymara speakers were less likely to use MCM (compared with Spanish speakers). The majority of these speakers belong to indigenous ethnical populations, which maintain ancestral behaviors on specific territories [52]. Quechua and Aymara are the primary Peruvian native languages; however, there are others such as Ashaninka, Awajun and Shipibo, etc. Furthermore, we believe that indigenous language speakers were experiencing a prior stage of the demographic transition; however, we did not find studies supporting this hypothesis. Likewise, speaking Quechua or Aymara was associated with a higher prevalence of mistreatment in health services [53]. In addition, due to their customs they are prone to use TCM, which are ineffective [54]. Although Spanish is the most spoken language in Peru, MINSA must guarantee access to FP information for native speakers. Its approach proposes interculturality, integrity, and social inclusion [2]; however, this may not be enough.

### Inequalities analysis

The higher the wealth index, the higher the prevalence of MCM utilization. However, the inequality analysis, at a nationwide level, indicated the presence of inequalities (but in small magnitude). However, when this analysis was stratified by area of residence, we found that rural areas had a higher magnitude of inequalities, even more than at the national level. This may be because the public health care system is oversaturated and faces expenditure shortages, subsequently, there is a high out-of-pocket spending on FP, particularly in rural areas [55], where health resources are scarce. Moreover, the majority of people living in rural areas are indigenous, and they typically use TCM [23]. Governmental social programs, such as the FP program, are of paramount importance for reducing inequality gaps of MCM utilization. Overall, the low magnitude of inequalities is the result of MINSA’s continued efforts to universally provide MCM.

### Spatial analysis

Common sociodemographic factors may underlie the observed spatial patterns in the regions with the lowest MCM use as the spatial distribution was clustered. Huancavelica, Cajamarca, and Puno are among the 10 poorest departments in our country: Huancavelica is the poorest [56]. In addition, Cajamarca and Puno are the least urbanized departments [57]. Women from rural areas are less educated and the majority of their health facilities are remote and poorly equipped [58, 59]. Furthermore, the time to health-care facilities was estimated to be 5.3 times longer in rural settings than in urban settings [60]. Differences in education are also indicated by the use of MCM. In fact, school attendance in Huancavelica, La Libertad, and Cajamarca is low [61]. There was also intra-departmental heterogeneity in the use of MCM. These geographic disparities may also be attributed to multiculturalism.

### Implication for policy and research

Several strategies must be implemented to improve Peruvian women’s access and use of MCM. Although decentralization is challenging, it can be achieved through efficient allocation of health resources. Health facilities and providers should be placed in rural areas, especially in native communities. Besides, the multicultural approach should be continued, and joint work between social actors and health workers should be boosted.

Unfortunately, most MCM are obtained from private health care providers [55, 62]. Therefore, social health insurance must enlarge its coverage, which must be accompanied by the inclusion of comprehensive FP strategies. In addition, this should encompass the expansion of MCM options and the improvement of sex education programs and counseling services among sexually active people, particularly targeted at all young people. Sexual education policies and programs should be developed on the basis of evidence-based thesis based on modern adolescent development theories and ecological models [63]. Furthermore, it is essential to ensure sufficient well-trained health providers nationwide.

The reduction of nonfinancial barriers is crucial. This could be remedied by establishing multi- and cross-sectoral efforts, such as the implementation of health centers in remote locations, improving of highways and roads, and improving FP services.

### Strengths and limitations

This study has several limitations. First, the DHS did not specify whether all women included in its survey were sexually active at the time of the interview. As the Peruvian DHS collects information from women 12 years and over, we included only those women who were of reproductive age. Second, we used a secondary database and thus had no data quality control. However, DHS interviewers received training courses and employed rigorous procedures for data quality control. Third, as it is a secondary database, there were interesting variables regarding the partner or the family that were excluded in the DHS. Fourth, due to the cross-sectional design, causality cannot be determined. Fifth, although the Peruvian DHS lacks a direct measure of socioeconomic status, we used an asset-based wealth index as a proxy variable, which is suitable for inequality studies in the absence of a direct measure [64]. However, to the best of our knowledge, this is the first study assessing inequalities and spatial distribution in the use of MCM among Peruvian women. Our results are derived from a large sample size, which implies a great statistical power and representativeness at the national level. In addition, the standardized definition of our outcome allows us to compare our results with other studies. Besides, we calculated ECI, which satisfy some shortcomings of the traditional CI [35].

## Conclusion

Two out of five Peruvian women of reproductive age used MCM. The use of MCM was directly associated with younger women’s age, younger age at first sexual intercourse, and being married or cohabitant, among other factors. However, it was inversely associated among those speaking Quechua or Aymara. No substantial inequality was found in MCM utilization at national level; however, it was higher in rural areas. The prevalence of use of MCM was heterogeneous at the intra- and inter-departmental level. Those departments located in the south, south-east, and north-east had the lowest prevalence of MCM utilization. Therefore, it is paramount to tackle nonfinancial barriers through multi- and cross-sectoral efforts and continue to universally provide MCM.

## Data Availability

The database is freely available and in the public domain. It can be found on the website "Microdatos" of the INEI in the survey section, where it is named as ENDES. (http://iinei.inei.gob.pe/microdatos/).

## Abbreviations

FP: Family planning
CM: Contraceptive methods
TCM: Traditional contraceptive methods
MCM: Modern contraceptive methods
HECM: Highly effective contraceptive methods
MINSA: Ministry of Health
DHS: Demographic and Health Survey
INEI: National Institute of Statistics and Informatics
WHO: World Health Organization
CC: Concentration curve
CI: Concentration index
AUC: Area under the curve
ECI: Erreygers’ normalized concentration index
95% CI: 95% Confidence interval
